# The Atlanta Society of Medicine

**Published:** 1890-06

**Authors:** 


### THE ATLANTA SOCIETY OF MEDICINE.

During the last few months the attendance at the meetings of the Atlanta Society of Medicine has been very meager. The Society which has done so much for the profession in Atlanta seems to be languishing. There must be some cause for this apathy, and that cause should be removed and the remedies applied. Let each officer and member be on hand at every meeting, prepared to take part in the discussions and report the interesting cases with which he has met, and the Society will soon be flourishing like a green bay tree.

We need and must have a better hall in which to hold our meetings.

The "Southern Surgical and Gynecological Association" will meet here in November, and each member of the profession in Atlanta must feel a pride in making that meeting a success. Through the Atlanta Society of Medicine must come that concert of action which is the *sine qua non* to a successful meeting.

We hope the members will keep in mind the nights of meeting—the first and third Tuesday nights of each month—and be on hand promptly at half past seven o'clock.

